# Emerging Importance of Helicases in Plant Stress Tolerance: Characterization of *Oryza sativa* Repair Helicase XPB2 Promoter and Its Functional Validation in Tobacco under Multiple Stresses

**DOI:** 10.3389/fpls.2015.01094

**Published:** 2015-12-16

**Authors:** Shailendra Raikwar, Vineet K. Srivastava, Sarvajeet S. Gill, Renu Tuteja, Narendra Tuteja

**Affiliations:** ^1^Plant Molecular Biology Group, International Centre for Genetic Engineering and BiotechnologyNew Delhi, India; ^2^Stress Physiology and Molecular Biology Lab, Centre for Biotechnology, Maharshi Dayanand UniversityRohtak, India; ^3^Amity Institute of Microbial Technology, Amity UniversityNoida, India

**Keywords:** agroinfiltration, rice, helicases, *OsXPB2* promoter, abiotic stress, tobacco

## Abstract

Genetic material always remains at the risk of spontaneous or induced damage which challenges the normal functioning of DNA molecule, thus, DNA repair is vital to protect the organisms against genetic damage. Helicases, the unique molecular motors, are emerged as prospective molecules to engineer stress tolerance in plants and are involved in nucleic acid metabolism including DNA repair. The repair helicase, XPB is an evolutionary conserved protein present in different organisms, including plants. Availability of few efficient promoters for gene expression in plants provoked us to study the promoter of XPB for better understanding of gene regulation under stress conditions. Here, we report the *in silico* analysis of novel stress inducible promoter of *Oryza sativa XPB2 (OsXPB2)*. The *in vivo* validation of functionality/activity of *OsXPB2* promoter under abiotic and hormonal stress conditions was performed by *Agrobacterium*-mediated transient assay in tobacco leaves using OsXPB2::GUS chimeric construct. The present research revealed that *OsXPB2* promoter contains cis-elements accounting for various abiotic stresses (salt, dehydration, or cold) and hormone (Auxin, ABA, or MeJA) induced GUS expression/activity in the promoter-reporter assay. The promoter region of *OsXPB2* contains CACG, GTAACG, CACGTG, CGTCA CCGCCGCGCT cis acting-elements which are reported to be salt, dehydration, cold, MeJA, or ABA responsive, respectively. Functional analysis was done by *Agrobacterium*-mediated transient assay using agroinfiltration in tobacco leaves, followed by GUS staining and fluorescence quantitative analyses. The results revealed high induction of GUS activity under multiple abiotic stresses as compared to mock treated control. The present findings suggest that *OsXPB2* promoter is a multi-stress inducible promoter and has potential applications in sustainable crop production under abiotic stresses by regulating desirable pattern of gene expression.

## Introduction

Essentially vital for all living organisms, the unique molecular motors, helicases unwind the duplex nucleic acids (i.e., DNA, RNA, or RNA-DNA hybrid) by using the free energy of ATP-binding/hydrolysis. Helicases remains present everywhere during the processing of nucleic acid in the cell and also emerged as potential candidate molecules for engineering abiotic stress tolerance in plants. Environmental cues continuously threaten the genomic integrity of all living organisms therefore in order to maintain the integrity of genome almost all the organisms throughout evolution contain robust DNA repair and recombination pathways to repair/remove or to tolerate lesions (Singh et al., 2011). Recent helicase research supports the potential of DNA/RNA helicases to counteract the adverse effect of various abiotic stress factors (Gill et al., 2014). *OsXPB2* is a member of highly conserved helicase super family 2 (SF2), in eukaryotes and it plays a vital role in DNA metabolism such as transcription and repair (Umate et al., 2011). XPB also known as ERCC3 and RAD25 is a 3′–5′ DNA helicase and it is an essential subunit of the eukaryotic basal transcription factor complex TFIIH [contains seven subunits (XPB, XPD, p62, p52, p44, p34, and TTDA)] (Schaeffer et al., 1993). XPB facilitates initiation of RNA polymerase II transcription and nucleotide excision repair (NER) by unwinding dsDNA around a DNA lesion. It has been reported that helicases play important roles in cell metabolic processes, including plant growth and development (Ribeiro et al., 1998; Costa et al., 2001). Various helicases have been known to function in providing abiotic stress tolerance to plants and few of them like *PDH45, MCM6*, and *p68* have been reported to contain stress inducible promoters (Sanan-Mishra et al., 2005; Luo et al., 2009; Dang et al., 2011a,b; Tajrishi and Tuteja, 2011; Gill et al., 2013; Tuteja et al., 2013; Banu et al., 2014). Therefore, exploitation of stress inducible promoters of candidate helicase genes can further complement the stress tolerance potential of crop plants.

In the present scenario, the *in silico* analysis of sequenced plant genome has become a routine to study and predict the promoter sequences (upstream of the 5′ end of the gene) and their contributing cis-acting elements. However, the demonstration of promoter activity is essential in order to confirm the functions of putative cis-elements. It is well-known that inducible promoters have broad biotechnological applications in the regulation of stress-related genes that are activated as a result of abiotic and biotic stresses (Kasuga et al., 1999; Oettgen, 2001). The inducible plant promoters based on their responsiveness, can be categorized as responsive to endogenous signals (plant hormones), external stimuli (biotic and abiotic stresses), and chemical stimuli. The promoters harbor various cis-regulatory elements and play vital role in the plant gene expression and regulation. Gene regulation can occur during different stages of gene expression and the most important point of control is RNA transcription. The promoter of Cauliflower mosaic virus (CaMV) 35S and its derivatives are used frequently for constitutive expression of transgene in plants and to achieve higher transgene expression (Odell et al., 1985; Battraw and Hall, 1990). However, the constitutive expression of functional genes/transcription factors in genetically engineered plants sometimes results in undesirable phenotype like growth inhibition or significant yield penalty (Capell et al., 1998; Liu et al., 1998; Kasuga et al., 1999; Hsieh et al., 2002). Therefore, the inducible promoters which can drive the expression of foreign genes under specific stresses can be of prime importance in engineering tolerance potential of crop plants (Kasuga et al., 1999). These inducible and tissue-specific promoters are central to the study of gene regulatory networks in plant (Huda et al., 2013; Oettgen, 2001). Different helicases like *PDH45, MCM6, SUV3, p68*, and *BAT1* are shown to be upregulated by abiotic stresses including salinity, dehydration, wounding, and low temperature and their overexpression conferred stress tolerance in plants (Sanan-Mishra et al., 2005; Tran et al., 2010; Tuteja et al., 2013, 2014a,b; Manjulatha et al., 2014).

Helicases are an intriguing aspect of the plant response to various stress factors but their potential has so far been poorly explored. Therefore, the functional validation of the upstream regulatory part or promoter of the DNA repair helicase *OsXPB2* gene is important for understanding its regulation under stress conditions. Thus, the isolation and functional characterization of *OsXPB2* promoter with respect to abiotic stresses and hormonal treatments may be of potential importance for engineering stress tolerance. The results presented in this report suggest that *OsXPB2* promoter can be a convincing tool that can be used as stress-inducible promoter for engineering crops with higher tolerance against abiotic stresses.

## Materials and methods

### *In silico* analysis of promoter

The 1000 bp promoter sequence upstream of the start codon of the *OsXPB2* gene (ID: Os01g49680; http://rice.plantbiology.msu.edu/) was retrieved from the rice genome database and *cis*-elements in the promoter were analyzed using PLANTCARE (http://bioinformatics.psb.ugent.be/webtools/plantcare/html/) and PLACE (http://www.dna.affrc.go.jp/PLACE/) database.

### Amplification of *OsXPB2* promoter and development of chimeric promoter-reporter construct

**Figure 1:**
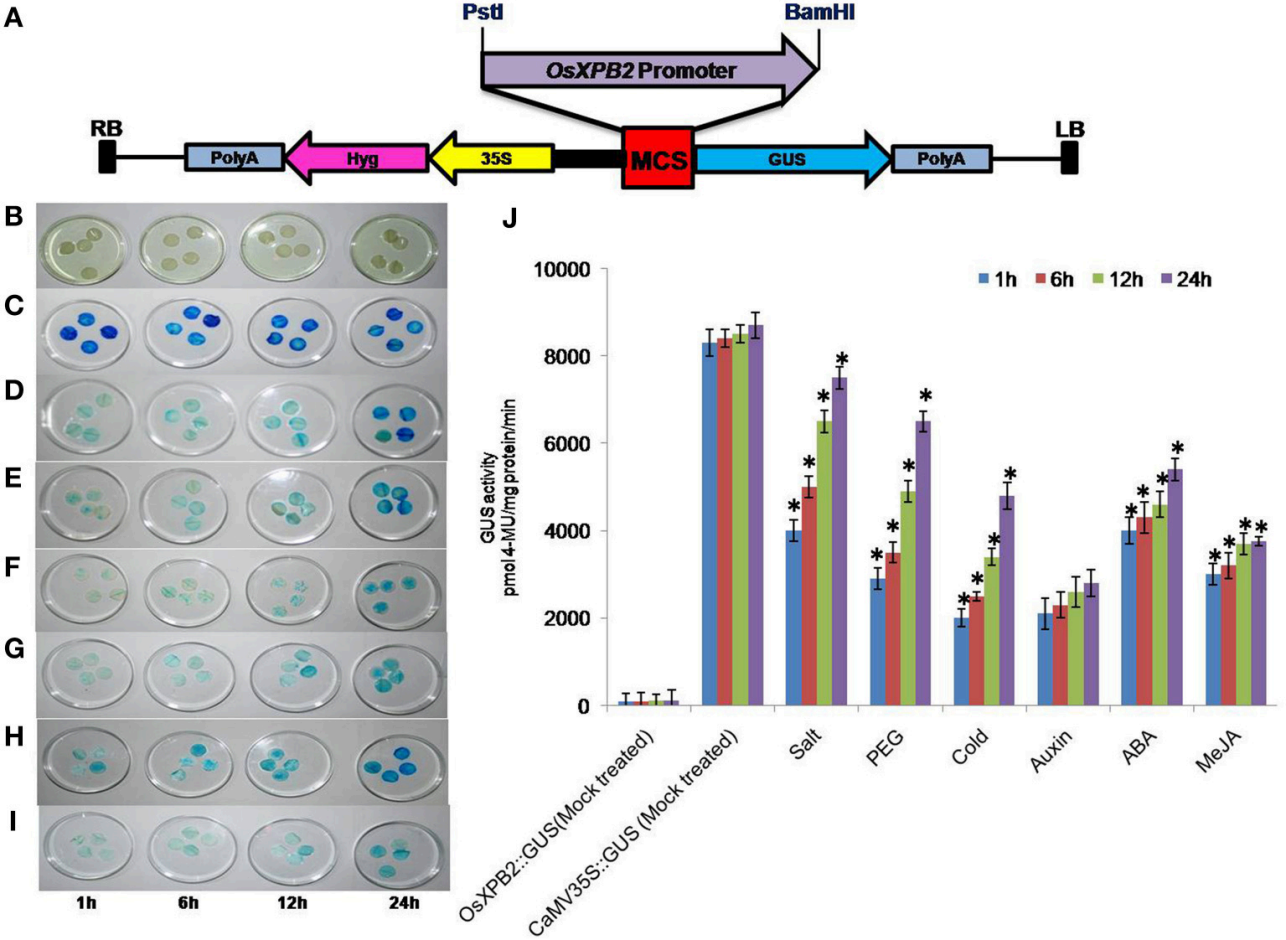
**(A)** Schematic representation of *OsXPB2* promoter cloned in pCAMBIA1391Z vector (promoter less vector) at PstI and BamHI sites for measuring GUS activity and agro-infiltration. **(B–J)** time course of *OsXPB2* promoter-*GUS* expression analysis in the agro-infiltrated tobacco leaves in response to abiotic stress [200 mM salt, 20% PEG, cold (4°C) stress] and phytohormones [MeJA (10 μM), ABA (5 μM), and auxin (10 μM)]. Histochemical GUS staining of *OsXPB2* promoter::GUS treated with water (mock treated) **(B)**, *CaMV35S*::GUS water (mock treated) **(C)**, *OsXPB2*::GUS NaCl **(D)**, PEG **(E)**, cold **(F)**, Auxin **(G)**, MeJA **(H)**, ABA **(I)**, comparison of GUS activity determined in protein extracts (*in vitro*) **(J)**. Data of four independent agro-infiltrated leaves were measured, and each experiment was replicated four times. Error bars on the graphic represent (±SD). ^*^P < 0.05 differ significantly from their respective controls according to Student's paired *t*-test.

Genomic DNA was isolated from the leaves of *Oryza sativa* (Var. IR 64) by CTAB method and 30 × dilution of genomic DNA was used as template for the amplification of *OsXPB2* promoter. Sequences of DNA adaptors and primers used for promoter amplification are *OsXPB2*FW: AACTGCAGAGACCCAGTGAAGCCAACACCCATTA, *OsXPB2*RV: ATGGATCCAACAT GGC CGG AAG CCC TGG AGC. The amplified fragment was cloned into pJET2.1 vector (Thermo Scientific). Subsequently the promoter was cloned into pCAMBIA-1391Z (promoter less vector) at PstI and BamHI restriction sites (Figure 1A, Supplementary Figure 1). The *OsXPB2* promoter cloned in pCAMBIA-1391Z was transformed in *Agrobacterium tumefaciens* (LBA4404) and confirmed by colony PCR using *OsXPB2* promoter specific primers.

### *Agrobacterium*-mediated transient assay

*Agrobacterium*-mediated transient assay was performed to study the expression of *OsXPB2* promoter using the method described by Yang et al. (2000). Fully expanded leaves of tobacco (*Nicotiana tobaccum* cv. USA) plants were agro-infiltrated by using 500 μl of bacterial suspension with 1 ml syringe into the abaxial surface of intact leaf. After 3 days, leaves were used for the stress and mock treatment analysis.

### Stress treatments

Agro-infiltrated leaf discs were soaked in petri dishes filled with 200 mM NaCl, 20% PEG (Polyethylene glycol), 5 μM ABA, 10 μM MeJA, or 10 μM NAA, respectively, and incubated for 1, 6, 12, or 24 h at room temperature. For cold stress, agro-infiltrated leaf discs were incubated at 4°C and the samples were collected at 1, 6, 12, and 24 h. Similarly, CaMV35S::GUS fusion construct transformed leaf discs were treated with H_2_O and used as mock treated control.

### Histochemical GUS staining and GUS activity quantification

GUS histochemical staining was performed using the method described earlier (Jefferson et al., 1987). The protein extraction (Bradford, 1976) and GUS fluorometric analysis was done using the method described earlier (Huda et al., 2013).

### Statistical analysis

Statistically significant differences between mean values were analyzed by Student's *t*-test (*P* ≤ 0.05).

## Results

### Isolation of *OsXPB2* promoter from rice and analysis of *CIS*-acting elements

*OsXPB2* promoter was amplified using promoter specific primer pairs as described earlier (Supplementary Figures 1A–C). Cis-acting elements present in the *OsXPB2* promoter region as identified by *in silico* analysis are listed in Table 1. The promoter region has a transcription start site TATA (TACAAA, consensus TTCC) and CCAAT box at position −55 and −61 base pair, respectively (Table 1). The sequence analysis suggests that several cis-elements including defense and stress responsiveness (TC-rich repeats), early responsive to dehydration (ABRELATERD1), dehydration responsive elements (CBFHV), heat shock protein responsive element (CCAATBOX1), cold response (MYCCONSENSUSAT), and element responsive to water stress (MYBCORE) are present in the *OsXPB2* promoter sequence (Table 1). The sequence also contains hormone responsive cis-acting elements like MeJ responsive CGTCA-motif, GCCCORE ethylene responsive element, GT1CONSENSUS SA response element and ABA responsive elements (e.g., Motif IIb). BS1EGCCR and skn-1 motifs are also identified to be associated with vascular tissue specificity and endosperm expression, respectively (Table 1). The cis-regulatory elements such as meristem specific (CCGTCC-box) element, wounding and pathogen response (box-s) element, phloem specific (RGATAOS), guard cell specific (TAAAGSTKST1), and mesophyll expression elements (CACTFTPPCA1) are also present in the sequence (Table 1).

**Table 1:** **Predictions of cis-elements present in *OsXPB2* promoter using PLANT CARE and PLACE database analysis**.

**Element**	**Position**	**Database ID**	**Strand**	**Expected function**
CCGTCC-box	427	PC	−	Meristem specific activation
CGTCA-motif	811	PC	+	MeJA-responsiveness
TGACG-motif	628	PC	+	MeJA-responsiveness
Skn-1_motif	627	PC	−	Endosperm expression
Circadian	204	PC	+	circadian control
AC-1	119	PC	+	Enhanced xylem expression and repressed phloem
CAAT-box	60	PC	+	Promoter and enhancer regions
TC-rich repeats	52	PC	+	Defense and stress responsiveness
CACTFTPPCA1	517	(P)S000449	+	Mesophyll expression module
CGACGOSAMY3	718	(P)S000205	+	Expression during sugar starvation
box S	459	PC	+	Elicitation; wounding and pathogen response
ABRELATERD1	46	(P) S000414	−	Early responsive to dehydration
ARFAT	824	(P) S000270	−	Auxin response factor(ARF)
ASF1MOTIFCAMV	629	(P) S000024	+	ASF-1 binding site” in CaMV 35S promoter
BS1EGCCR	882	(P) S000352	+	Vascular expression
CBFHV	49	(P) S000497	−	Dehydration-responsive element (DRE)
CCAATBOX1	165	(P) S000030	+	Heat shock protein genes
GCCCORE	612	(P) S000430	+	Ethylene-responsive element
	921			
	950			
	961			
GT1CONSENSUS	861	(P) S000198	+	SA-inducible gene expression
MYB2CONSENSUSAT	70	(P) S000409	+	Dehydration-response
MYBCORE	152	(P) S000176	+	Responsive to water stress
MYCCONSENSUSAT	211	(P) S000407	+	Cold response
	384			
	567			
PREATPRODH	647	(P) S000450	−	Hypoosmolarity-responsive element
RGATAOS	254	(P) S000191	+	Phloem-specific
TAAAGSTKST1	863	(P) S000387	+	Guard cell-specific
TATCCACHVAL21	42	(P) S000416	+	GA response
WRKY71OS	629	(P) S000447	+	Transcriptional repressor of the gibberellins signaling pathway
Motif IIb	961	PC	+	Abscisic acid responsive element

### Cloning of *OsXPB2* promoter and its activity in tobacco leaves

The *OsXPB2* promoter was cloned into pCAMBIA-1391Z and the clones were confirmed by PCR and restriction analysis (Supplementary Figures 1A–C). Different stress responsive cis-elements are shown in the *OsXPB2* promoter sequence (Supplementary Figure 2). The fusion construct *OsXPB2*::*GUS* was transiently expressed in tobacco leaves and it was used to check the promoter inducibility under different abiotic and hormonal stress conditions at different time points to study time course of GUS activity. To check whether the isolated promoter region of *OsXPB2* possesses active promoter functions, the tobacco leaves agro-infiltrated with *OsXPB2*::GUS or CaMV35S::GUS (as control) were mock treated (Figures 1B,C). There was no blue color development in the mock treated *OsXPB2* promoter and very low level of GUS expression and activity was recorded (Figures 1B,J). The CaMV35S::GUS was also given mock treatment and an intense blue coloration developed at different time points suggesting very high GUS activity (Figures 1C,J). The activity of *OsXPB2* promoter was analyzed under different abiotic stress (Salt, PEG, or cold) conditions by transient assay (Figures 1D–F). Histochemical staining revealed that the GUS expression increased at 12–24 h as compared to 1 and 6 h (Figure 1D). The effect of PEG stress varied for the *OsXPB2* promoter; the blue staining was detected at 1–6 h of stress treatment, but the intensity of the blue color gradually increased from 12 to 24 h, and similar trend was also observed in the quantitative GUS activity (Figures 1E,J). Under cold stress treatment at the early time period 1–6 h, slight blue coloration was detected and increase in the blue color intensity was present at 24 h (Figures 1F,J). These results reveal that *OsXPB2* promoter is a stress inducible promoter and it mainly responds to osmotic and cold stresses.

Leaf disks were also incubated with different hormones (NAA, ABA, or MeJA) and differential pattern in GUS activity was noted (Figures 1G–I). The GUS expression driven by *OsXPB2* promoter under NAA treatment showed moderate blue color at 1–12 h and slight increase was noted at 24 h and the corresponding GUS activity was also recorded (Figures 1G,J). It is interesting to note that in the ABA treatment the blue color was intense in the initial time period and it sustained up to 24 h and the GUS activity recorded was also high (Figures 1H,J). Furthermore, in the MeJA treated leaves the blue color developed but the variation in the blue color did not differ much up to 24 h, and the corresponding GUS activity was recorded (Figures 1I,J). The observed variation in GUS expression levels may be due to difference in response of cis-acting elements of *OsXPB2* promoter.

## Discussion

Helicases, the motor proteins have vast potential as modulators of stress responses in plants. The new emerging role of helicases in engineering plant abiotic stress tolerance has encouraged studying the associated promoters for better understanding of gene regulation under stressful conditions. At present, constitutive and inducible promoters are widely used for the expression of candidate genes and their functional analysis. However, the constitutive expression of transgene may lead to homology-dependent gene silencing. Therefore, the exploitation of inducible promoters may be a vital tool for spatial and temporal gene expression under stress. In this study, we have presented important information regarding the complex regulation of rice helicase promoter in response to different abiotic stresses. Recent report regarding the function of *OsXPB2* gene in DNA damage and the concomitant activation of TC-NER pathway in response to γ-radiation and salinity stress emphasizes the importance of helicases in abiotic stress tolerance (Macovei et al., 2014). Therefore, the identification and functional validation of cis-elements is crucial in understanding the regulation of promoter and its possible exploitation in transgenic research. It is well-established that the putative regulatory elements in plant promoters can be easily identified using *in silico* analysis (Pujade-Renaud et al., 2005; Wei-Min et al., 2005; Huda et al., 2013). The analysis of cis-regulatory elements present in the promoter regions have received special attention as they provide insights into gene regulation and plant signaling under stress conditions. Further, *Agrobacterium*-mediated transient expression assay is a widely accepted method for *in vivo* quantitative analysis of plant promoters and cis-element/trans-factor interactions (Yang et al., 2000). The analysis of the expression of GUS reporter gene in *OsXPB2*::GUS revealed abiotic and hormonal regulation of GUS expression.

Brosché et al. (2002) reported that the promoter of XPD helicase of *Arabidopsis thaliana* contains multiple cis-elements [ACGT, ACCTA, H-box, myeloblastosis (Myb), Myb recognition element (MRE), SET binding factor 1 (SBF-1) and TCA-element, salicylic acid-responsive element] and has implication in light regulation and in UV stress response. It has also been reported that *AtXPD* gene was among some DNA repair genes that are hypomethylated in the promoter region (Boyko et al., 2010). Hypomethylation was reported to be correlated with permissive chromatin histone modification and increased *AtXPD* expression (Boyko et al., 2010). The significance of few cis-regulatory elements like G-box and ABREs combinations have also shown that stress-responsive genes are regulated by multiple transcription factors (Abe et al., 1997; Liu et al., 2014; Wang et al., 2014). Therefore, functional analysis of cis-regulatory elements is crucial to understand the regulatory gene networks in stress-responsive pathways. Our present study demonstrates the presence of different stress responsive cis-elements in *OsXPB2* promoter that are associated with tissue-specific expression, meristem specific, endosperm specific expression, defense and stress responsiveness, vascular expression, phloem specific, guard cell specific, and mesophyll expression module. The presence of these tissue-specific expression regulatory elements indicates the association of *OsXPB2* gene to a wide range of cellular processes which still requires validation. In addition, the *in silico* analysis of *OsXPB2* promoter suggests the presence of salt or dehydration responsive cis-acting elements in the sequence.

*In vivo* analysis of *OsXPB2*::GUS construct revealed that GUS expression was induced by different abiotic stresses and *OsXPB2* promoter was able to drive GUS expression when agro-infiltrated in tobacco leaves treated with NaCl, PEG or cold stress. The presence of multiple copies of the NAC like element (5′-CACG-3′) in the upstream region of *OsXPB2* gene might be responsible for salt induced expression. Tran et al. (2004) reported that NAC–type transcription factors regulate salt responsive genes in an ABA-dependent manner. Salt stress was also shown to induce several NAC genes in rice (Hu et al., 2006). It has been reported that OsNAC5 salt inducible NAC transcription factor which binds to the NAC recognition core sequence (CACG) of *OsLEA3* promoter, when overexpressed, showed improved salt tolerance (Takasaki et al., 2010).

Furthermore, the PEG and ABA treatment leads to higher GUS activity. A significant increase in ABA levels has been observed in response to dehydration stress. Previous reports also support that most dehydration-inducible genes are induced by ABA (Chandler and Robertson, 1994; Shinozaki et al., 2003). The upstream region of *OsXPB2* gene also contains multiple cold responsive elements, e.g., MYCCONSENSUSAT (CACGTG). Chinnusamy et al. (2003) reported that cold stress induced ICE1 binds to MYC cis-elements of the CBF promoter in *Arabidopsis* and it induces the expression of CBF, which regulates the COR genes and imparts cold acclimation.

Plant hormones are known to mediate the defense processes against pathogenic attack and herbivors (Ohshima et al., 1990). Furthermore, phytohormones like salicylate, jasmonates, and ethylene are reported to be involved in plant responses to various stresses (Ohshima et al., 1990). It is well-established that phytohormone auxin regulates several physiological processes such as apical dominance, shoot elongation, lateral root initiation, vascular differentiation, embryo patterning etc. (Davies, 1995) and enhances the transcription of various genes (Aux/IAA, GH3, and SAUR gene family members) (Abel and Theologis, 1996). In the present study, we have identified auxin responsive cis-regulatory elements in *OsXPB2* promoter sequence and high GUS expression was observed in the agro-infiltrated tobacco leaves. Jasmonates including MeJA and JA are also key signaling molecules for diverse developmental processes from seed germination to fruit ripening and senescence (Wasternack and Hause, 2002). The GCC or G-box elements, CGTCA motif and TGACG motif are required for MeJA-inducible expression of different genes. The role of JA in response to various abiotic stresses has been reported in a number of studies (Clarke et al., 2009; Yoon et al., 2009; Brossa et al., 2011). The analysis of GUS expression in response to MeJA indicates that the cis-elements present in *OsXPB2* promoter may have positive regulatory role toward stress tolerance.

In the present study, using histochemical analysis (qualitative and quantitative) we have demonstrated that the *OsXPB2* promoter is able to drive GUS reporter gene expression in response to abiotic stress and hormonal treatments. The cis-elements identified in *OsXPB2* promoter together with the data from *GUS* reporter gene expression profiles under different abiotic stresses, support that *OsXPB2* promoter is stress responsive. The transient assay results along with GUS fluorometric assay results show that the *OsXPB2* promoter triggers high levels of *GUS* expression under abiotic and hormonal treatment stresses. Our data collectively suggest that the *OsXPB2* promoter analyzed in the present study could be potentially used to drive transgenes based on its responsiveness to different abiotic stresses including the genotoxic stress for the crop improvement.

### Conflict of interest statement

The authors declare that the research was conducted in the absence of any commercial or financial relationships that could be construed as a potential conflict of interest.
