# Supplementary material for: Emerging Importance of Helicases in Plant Stress Tolerance: Characterization of Oryza sativa Repair Helicase XPB2 Promoter and Its Functional Validation in Tobacco under Multiple Stresses
Source: Front Plant Sci. 2015 Dec 16;6:1094. doi: 10.3389/fpls.2015.01094 (PMC4679908; doi:10.3389/fpls.2015.01094)
Supplement: Supplementary file 1 [file Presentation1.PPTX]

## Slide 1
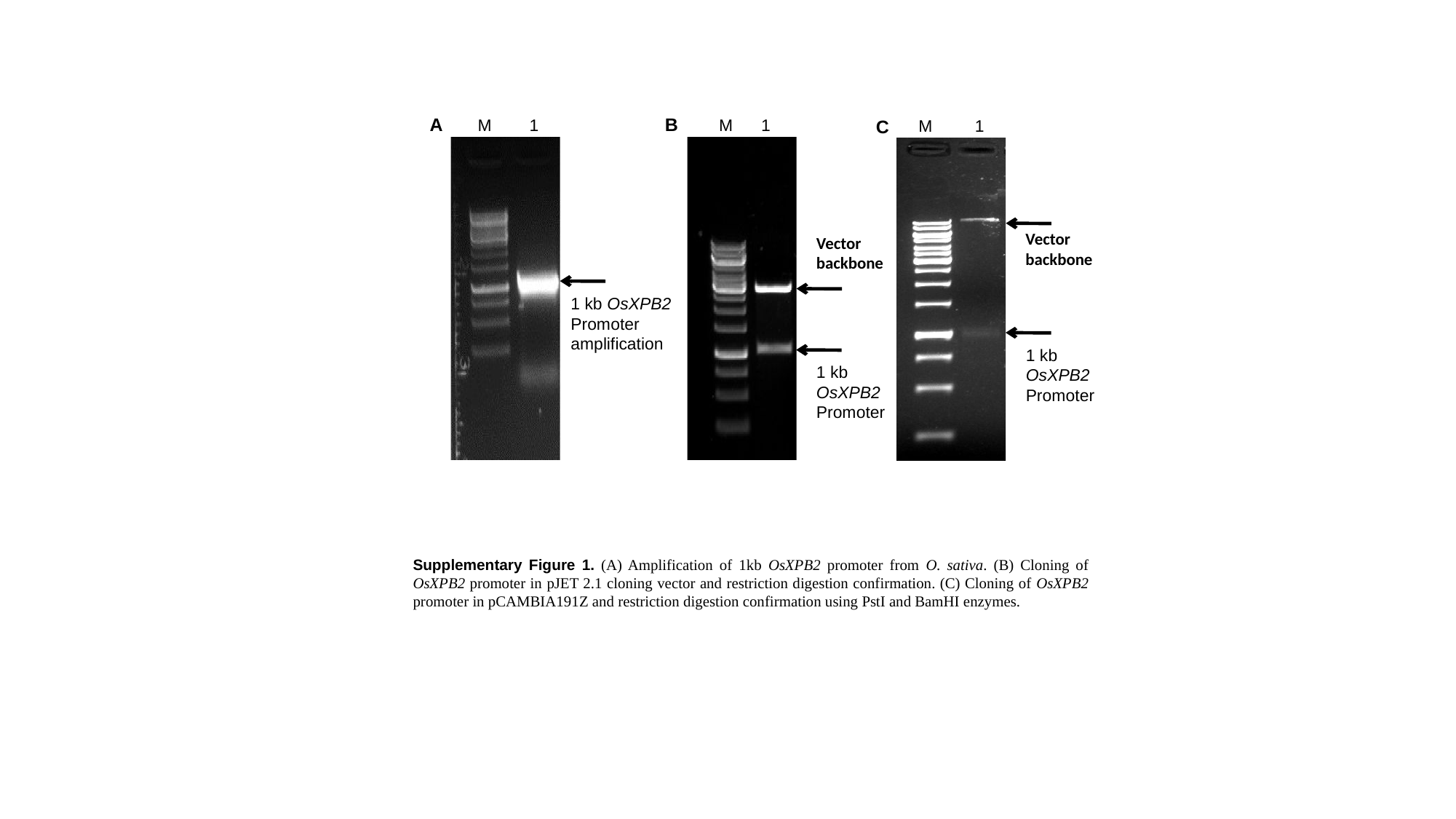

A
B
 M 1
 M 1
C
 M 1
Vector backbone
Vector backbone
1 kb OsXPB2 Promoter
amplification
1 kb OsXPB2 Promoter
1 kb OsXPB2 Promoter
Supplementary Figure 1. (A) Amplification of 1kb OsXPB2 promoter from O. sativa. (B) Cloning of OsXPB2 promoter in pJET 2.1 cloning vector and restriction digestion confirmation. (C) Cloning of OsXPB2 promoter in pCAMBIA191Z and restriction digestion confirmation using PstI and BamHI enzymes.
