# Supplementary material for: Emerging Importance of Helicases in Plant Stress Tolerance: Characterization of Oryza sativa Repair Helicase XPB2 Promoter and Its Functional Validation in Tobacco under Multiple Stresses
Source: Front Plant Sci. 2015 Dec 16;6:1094. doi: 10.3389/fpls.2015.01094 (PMC4679908; doi:10.3389/fpls.2015.01094)
Supplement: Supplementary file 2 [file Presentation2.PPTX]

## Slide 1
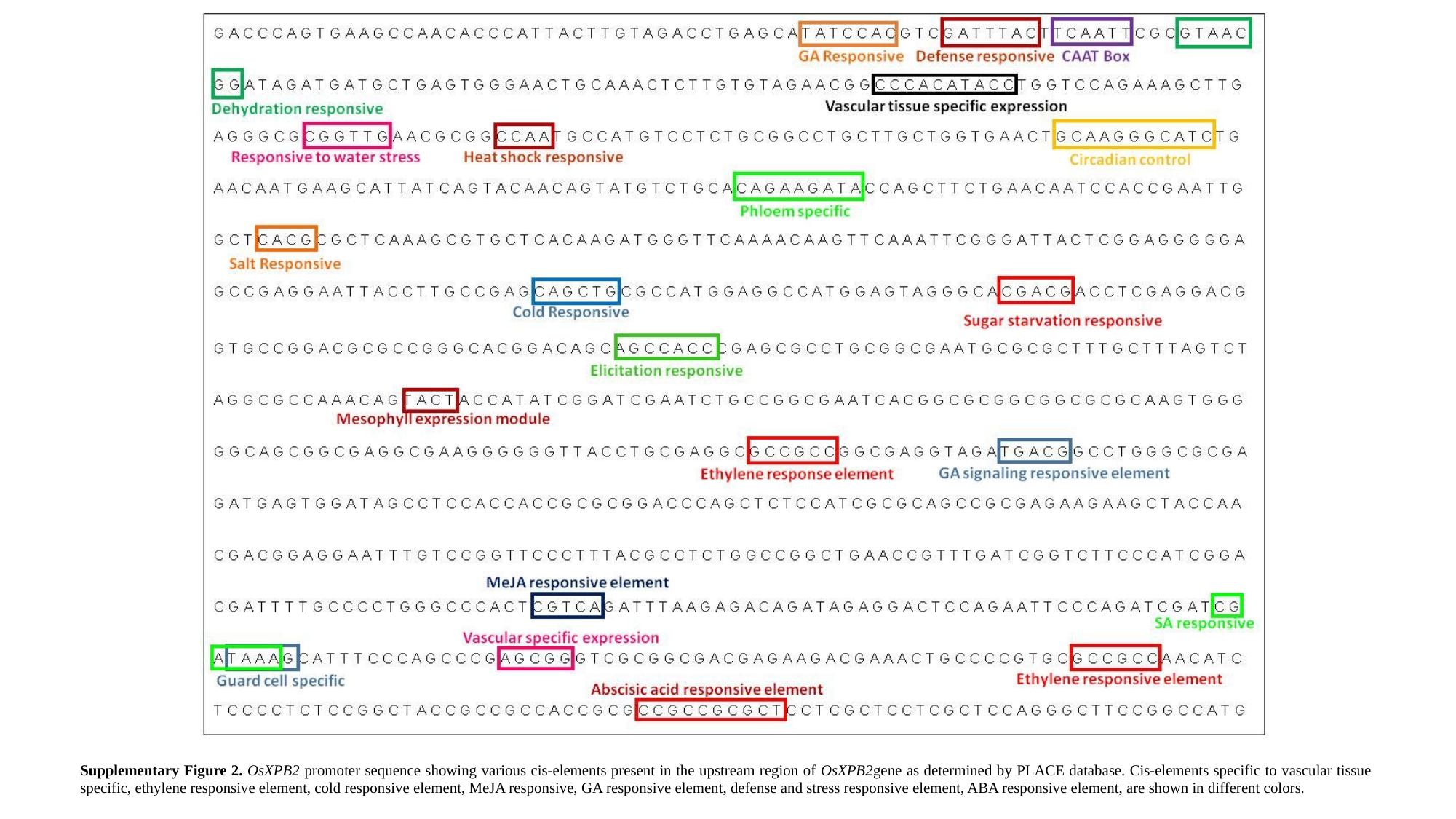

Supplementary Figure 2. OsXPB2 promoter sequence showing various cis-elements present in the upstream region of OsXPB2gene as determined by PLACE database. Cis-elements specific to vascular tissue specific, ethylene responsive element, cold responsive element, MeJA responsive, GA responsive element, defense and stress responsive element, ABA responsive element, are shown in different colors.
